# Alcoholic Liver Disease: Update on the Role of Dietary Fat

**DOI:** 10.3390/biom6010001

**Published:** 2016-01-06

**Authors:** Irina A. Kirpich, Matthew E. Miller, Matthew C. Cave, Swati Joshi-Barve, Craig J. McClain

**Affiliations:** 1 Division of Gastroenterology, Hepatology and Nutrition, Department of Medicine, University of Louisville School of Medicine, Louisville, KY 40202, USA; millerm25@udayton.edu (M.E.M.); matt.cave@louisville.edu (M.C.C.); swati.joshi-barve@louisville.edu (S.J.-B.); craig.mcclain@louisville.edu (C.J.M.); 2 Department of Pharmacology and Toxicology, University of Louisville School of Medicine, Louisville, KY 40202, USA; 3 Robley Rex Veterans Medical Center, Louisville, KY 40202, USA

**Keywords:** alcoholic liver disease, saturated and unsaturated dietary fat, oxidized dietary lipids, oxidized linoleic acid metabolites, gut microbiota

## Abstract

Alcoholic liver disease (ALD) spans a spectrum of liver pathology, including fatty liver, alcoholic steatohepatitis, and cirrhosis. Accumulating evidence suggests that dietary factors, including dietary fat, as well as alcohol, play critical roles in the pathogenesis of ALD. The protective effects of dietary saturated fat (SF) and deleterious effects of dietary unsaturated fat (USF) on alcohol-induced liver pathology are well recognized and documented in experimental animal models of ALD. Moreover, it has been demonstrated in an epidemiological study of alcoholic cirrhosis that dietary intake of SF was associated with a lower mortality rates, whereas dietary intake of USF was associated with a higher mortality. In addition, oxidized lipids (dietary and *in vivo* generated) may play a role in liver pathology. The understanding of how dietary fat contributes to the ALD pathogenesis will enhance our knowledge regarding the molecular mechanisms of ALD development and progression, and may result in the development of novel diet-based therapeutic strategies for ALD management. This review explores the relevant scientific literature and provides a current understanding of recent advances regarding the role of dietary lipids in ALD pathogenesis.

## 1. Introduction

Alcohol-associated health problems, including alcoholic liver disease (ALD), are a major medical problem within the United States and worldwide. ALD is an umbrella term for the alcohol-induced liver pathology, including fatty liver, alcoholic hepatitis, and cirrhosis, which may further progress to hepatocellular carcinoma. Importantly, clinically important ALD develops only in a subset of people who drink heavily. Alcoholic hepatitis occurs in approximately 10%–35% of chronic drinkers, and severe alcoholic hepatitis accounts for significant morbidity and mortality approaching 35%–45%. Approximately 10%–20% of heavy drinkers will develop cirrhosis [1,2,3]. To date, ALD prevention and treatment strategies have been generally ineffective, in part, due to lack of knowledge regarding the molecular mechanisms underlying ALD development and progression.

Accumulating evidence suggests that dietary factors, including dietary fat, along with heavy alcohol consumption, play critical roles in ALD pathogenesis. Indeed, the beneficial effects of dietary saturated fat (SF) and damaging effects of dietary unsaturated fat (USF, primarily corn oil/linoleic acid (LA) enriched) on alcohol-induced liver injury have been documented in experimental animal models of ALD [4,5,6,7,8,9,10]. Moreover, comparison of dietary fat intake in various countries with similar per capita alcohol consumption has demonstrated that dietary intake of SF was associated with lower mortality rates, whereas dietary intake of USF was associated with a higher mortality from alcoholic cirrhosis [11]. However, the underlying mechanism(s) by which different types of dietary fat potentiate or attenuate ALD are not completely determined. Understanding of how dietary lipids contribute to ALD pathogenesis will enhance our knowledge regarding the molecular mechanisms of ALD development and progression, and may help to identify novel dietary intervention strategies for ALD prevention/treatment as well as to explain why only some people who drink heavily develop clinically-relevant ALD. The present review summarizes the current knowledge and recent advances regarding the impact of dietary lipids on ALD pathogenesis, including the effects of different types of dietary fat on alcohol-mediated hepatic steatosis, injury, as well as intestinal permeability, endotoxemia, and gut microbiota changes.

## 2. Dietary Saturated and Unsaturated Fat: Role in ALD Pathogenesis

The critical role for the specific types of dietary fat in ALD pathogenesis has been demonstrated and extensively studied in different pre-clinical animal models of ALD using various sources of dietary lipids (e.g., beef fat, cocoa butter, corn oil, fish oil, palm oil, medium chain triglyceride (MCT) oil, Table 1). These diverse dietary lipids have several distinct characteristics, including differences in fatty acid composition, such as carbon chain length (e.g., short, medium and long chain fatty acids), and degree of saturation (e.g., saturated, mono- and polyunsaturated fatty acids, PUFAs, Table 2). In addition, total amount of saturated and unsaturated fatty acids, as well as other compounds (e.g., carotenoids, tocopherols, polyphenols, and glycerol) could also be quite variable between different types of dietary fat. Thus, lard, beef tallow, cocoa butter, and palm oil have a mixture of long-chain saturated fatty acids (mostly C18:0 and C16:0) and monounsaturated fatty acids (primarily C18:1 n9). Corn oil, safflower oil, and soybean oil contain large amount of PUFAs, with the C18:2 n6 as a major PUFA. All the fatty acids in MCT oil are less than 12 carbon saturated fatty acids, with the most abundant being C8:0 fatty acid. It is worth noting that fatty acids with different carbon chain lengths (e.g., long chain *vs.* medium chain fatty acids) have different metabolic and absorptive pathways. Unlike long chain fatty acids, medium chain fatty acids do not require incorporation into chylomicrons; they are absorbed directly from the diet into the hepatic portal vein. In addition, medium chain fatty acids do not need carnitine acyltransferase for their transport into the inner mitochondria membrane, facilitating rapid transport and oxidation [12]. It has been recently demonstrated that increasing the ratio of MCT oil (rich in medium chain saturated fatty acids) to corn oil (rich in long chain PUFAs) resulted in decreased hepatic steatosis and serum ALT levels in a rat model of non-alcoholic fatty liver disease (NAFLD). This was associated with increased peroxisome proliferator-activated receptor alpha (PPARα)-dependent hepatic fatty acid oxidation, and reduced membrane susceptibility to free radical attack [13].

The series of initial experimental studies from Nanji’s group showed that dietary SF attenuated, and dietary USF, specifically rich in corn oil, promoted alcohol-induced liver damage in experimental animals [14,15]. Moreover, dietary LA (a PUFA with two double bonds, 18:2 n-6), was required for the development of experimental ethanol (EtOH)-induced liver injury; and the severity of ALD was correlated with the amount of LA in the diet [16]. It has also been reported that rats fed EtOH and a diet rich in menhaden fish oil (which contains a large percentage of PUFAs with more than two double bonds) developed pathological changes, including fatty liver, necrosis, and inflammation, even more severe than those fed EtOH and a diet enriched in corn oil [17]. Importantly, dietary SF (e.g., palm oil or MCT oil) reversed the established experimental ALD in rats, and improved liver histologic changes despite continued intragastric EtOH administration [18]. The dose-dependent improvement of EtOH-induced liver pathology and oxidative stress was observed in rats fed alcohol when dietary SF (beef tallow and MCT oil) was substituted for USF (corn oil) at various levels [6] in a dose-dependent manner. Dietary supplementation with olive oil, which contains a rich variety of natural antioxidants (e.g., carotenoids, tocopherols, and polyphenols) protected against hepatic lipid peroxidation, leading to a decrease in liver injury in rats chronically exposed to EtOH [19]. A recently-published report has demonstrated that rats fed lard as a source for dietary SF exhibited decreased EtOH-induced hepatic fat accumulation; however, liver necrosis and fibrosis scores were increased in comparison to animals fed EtOH and a diet containing a combination of corn, olive, and safflower oils [20].

**Table 1:** The overall effects of different types of dietary fat on EtOH-mediated changes in the intestine and the liver *.

Animal Models	Dietary Regimen **	Significant Outcomes	References
Rats fed EtOH or control liquid diets with a Ritcher drinking tube for eight weeks.	CO + OO + SFO *vs.* L + SBO; 36%E	Hepatic steatosis and inflammation: CO + OO + SFO + EtOH > L + SBO + EtOH; Hepatic fibrosis: CO + OO + SFO + EtOH < L + SBO + EtOH	[20]
Rats fed intragastrically EtOH or control liquid diets for four weeks.	MCT *vs.* CO *vs.* FO; 25%E	Severity of liver pathology: FO + EtOH > CO + EtOH; MCT + EtOH (no pathological changes)	[15]
Rats fed intragastrically EtOH or control liquid diets for 10 weeks.	CO (USF diet) *vs.* BT + MCT (SF diet); 45%E	Liver steatosis, injury, and oxidative stress: USF + EtOH > SF + EtOH. SF protected rats from ALD in a dose-responsive fashion	[6]
Rats fed solid food and administered EtOH daily (IP) for six weeks	CO *vs.* CO + OO supplementation (5% *wt*/*wt*)	Liver injury, oxidative stress: CO + EtOH > CO + OO + EtOH	[19]
Rats fed *at libitum* EtOH or control liquid diets for eight weeks.	CO *vs*. CB *vs.* MCT; 30%E	Liver steatosis, macrophage activation, neutrophil infiltration, and hepatic endotoxin levels: CO + EtOH > CB + EtOH or MCT + EtOH; Serum endotoxin levels: CO + EtOH = CB + EtOH; CO + EtOH > MCT + EtOH	[7]
Mice fed intragastrically EtOH or control liquid diets for three weeks.	CO (USF diet), *vs*. Hydrogenated soya glyceride (12% palmitic and 85% stearic acids, SF diet); 35%E	Liver steatosis, injury, and oxidative stress: USF + EtOH > SF + EtOH	[8]
Mice fed *at libitum* EtOH or control liquid diets for four weeks.	CO *vs.* CB; 40%E	Liver injury and steatosis: CO + EtOH > CB + EtOH; Plasma adiponectin: CO + EtOH < CB + EtOH	[9]
Mice fed *at libitum* EtOH or control liquid diets for eight weeks.	CO (USF diet) *vs.* BT + MCT (SF diet); 40%E	Liver steatosis, inflammation, and injury: USF + EtOH > SF + EtOH. Intestinal inflammation, alterations in intestinal tight junctions, increased gut permeability and endotoxemia: USF + EtOH > SF + EtOH	[5,21]

Abbreviations: Beef Tallow, BT; Cocoa Butter, CB; Corn Oil, CO; IP, Intraperitoneal Injection; Fish (menhaden) Oil, FO; Olive Oil, OO; Lard, L; Medium Chain Triglyceride oil, MCT; Safflower Oil, SFO; Sunflower Oil, SnFO; Soybean Oil, SBO; %E, percent energy from fat. * The selected studies include different animal models of ALD (e.g., intragastric *vs*. *ad libitum* EtOH administration; rats *vs*. mice), and different dietary fat sources. ** The detailed fatty acid composition of fat sources is provided in the Table 2.

**Table 2:** Fatty acid composition of typical fat sources in experimental diets.

FFAs	Lard	Beef Fat (Tallow)	Cocoa Butter	MCT Oil	Corn Oil	Palm Oil	Fish Oil ^a^	Olive Oil	Safflower Oil	Soybean Oil
**Saturated FFAs**										
**C24:0**	-	-	-	-	-	0.1	-	0.1	0.1	0.3
**C22:0**	-	-	0.2	-	-	0.1	-	0.1	0.3	0.2
**C20:0**	-	0.1	1.2	-	0.1	0.3	-	0.4	0.4	0.3
**C19:0**	-	0.1	-	-		-	-	-	-	-
**C18:0**	14	21.6	36.4	-	2.2	4.4	2.1	2.6	2.3	3.9
**C17:0**	-	1.5	-	-	0.1	-	-	-	-	-
**C16:0**	26	25.5	25.1	-	-	43.8	13.0	12.1	6.1	10.8
**C15:0**	-	1.3	-	-	-	-	-	-	-	-
**C14:0**	2	3.3	-	-	-	1.1	11.6	-	-	0.1
**C12:0**	-	0.1	-	-	-	0.4	-	-	-	-
**C10:0**	-	0.1	-	23 (4 > 10:0)	-	0.1	-	-	-	-
**C8:0**	-	-	-	67 (6 < 8:0)	-	0.1	-	-	-	-
**Unsaturated FFA (Monounsaturated FFAs)**										
**C24:1 n9**	-	-	-	-	-	-	-	-	0.2	-
**C20:1 n9**	1	-	-	-	-	0.1	-	0.3	0.2	0.1
**C18:1 n9**	44	38.7	34.1	-	27.5	39.1	6.7	72.5	13.4	23.9
**C18:1 n7**	-	-	-	-	-	-	3.3	-	-	-
**C17:1**	-	0.7	-	-	-	-	-	0.2	-	-
**C16:1**	3	3.4	-	-	12.2	0.2	13.3	0.8	0.1	0.2
**C15:1**	-	0.2		-	-	-	-	-	-	-
**C14:1**	-	0.2	-	-	-	-	-	-	-	-
**Unsaturated FFAs (Polyunsaturated FFAs)**										
**C22:6 n3**	-	-	-	-	-	-	8.2	-	-	-
**C22:5 n6**	-	-	-	-	-	-	0.4	-	-	-
**C22:5 n3**	-	-	-	-	-	-	2.0	-	-	-
**C20:5 n3**	-	-	-	-	-	-	17.3	-	0.5	-
**C20:4 n3**							1.9			
**C20:4 n6**	-	0.4	-	-	-	-	0.7	-	0.5	-
**C18:3 n3**	-	0.6	0.2	-	0.9	0.3	-	0.6	0.3	7.8
**C18:2 n6**	10	2.2	2.8	-	57.0	10.2	1.1	9.4	76	52.1
**References**	[22]	[6]	[23]	[6]	[6]	[23]	[15]	[23]	[23]	[23]

**^a^**—fish (menhaden) oil (% by weight).

Based on early studies, the deleterious effects of dietary USF in comparison to protective effects of dietary SF were thought to be mediated through induction of lipid peroxidation and oxidative stress [6,15,24,25,26], elevated endotoxin levels, and associated increased production of pro-inflammatory cytokines [4,18,26]. A number of recent elegant studies have enhanced our knowledge regarding the molecular mechanisms by which dietary USF promotes/exacerbates while the dietary SF prevents/ameliorates ALD. For example, in comparison to dietary USF (corn oil), the beneficial effects of dietary SF (cocoa butter) in ALD was attributed to the modulation of the hepatic SIRT1-SREBP-1-histone H3 (Sirtuin—Sterol Regulatory Element Binding Protein-1c—histone H3) axis, resulting in suppression of genes encoding lipogenic enzymes [27], and the induction of adiponectin, the adipocyte hormone known to play a favorable role in ALD [9,28]. The increase in circulating adiponectin was associated with the activation of a set of hepatic signaling pathways mediated through AMP-activated protein kinase (AMPK), PPARα, and peroxisome proliferator-activated receptor gamma coactivator 1 alpha (PGC-1α), which, in turn, led to increased rates of fatty acid oxidation, prevention of hepatic steatosis, and attenuation of liver enzyme changes [9]. Another recently identified mechanism of how dietary lipids influence ALD development and progression is through hepatocyte nuclear factor-4α (HNF4α), a master transcription factor in the regulation of lipid metabolism. It has been reported that EtOH and dietary USF (corn oil) but not SF (MCTs) resulted in increased hepatic fat accumulation in rats in parallel with the decreased levels of HNF4α. The authors postulated that dietary MCTs attenuated alcohol-induced hepatic lipid accumulation, at least partially, through the prevention of HNF4α reduction [29]. Further, EtOH-mediated reductions in HNF4α in the intestine were associated with decreased levels of the intestinal tight junction (TJ) proteins and disruption of intestinal barrier integrity [30]. Impaired intestinal barrier integrity is one among several causal factors of alcoholic endotoxemia, a critical factor contributing to the ALD development and progression. There are several lines of evidence suggesting that different types of dietary lipids may differentially modulate EtOH-mediated intestinal barrier disruption, endotoxemia, and subsequent liver injury. Recent studies from our group have demonstrated that eight weeks of EtOH feeding significantly increase liver steatosis, inflammation and injury in mice fed EtOH and USF (corn oil) compared to mice fed EtOH and SF diet containing beef tallow and MCT oil [5]. Hepatic Toll-like receptor mRNA levels (TLR 1, 2, 3, 4, 7, 8, 9) were significantly increased compared to control in the livers of USF + EtOH fed animals, but not in the livers of the SF + EtOH group. In parallel with liver injury, significantly increased gut permeability and elevated endotoxemia were observed in response to USF + EtOH but not SF + EtOH [5]. Intestinal inflammation was positively correlated with the USF + EtOH triggered disruption of the intestinal TJs. Importantly, USF diet alone resulted in down-regulation of intestinal TJ protein mRNA expression compared to SF. Alcohol further suppressed TJ proteins in USF + EtOH, but not in the SF + EtOH group. Additionally, USF + EtOH, but not SF + EtOH, resulted in alterations of the intestinal mucus layer and intestinal antimicrobial defense [21].

Importantly, the beneficial effects of dietary SF on EtOH-induced liver injury depend on the dietary fatty acid chain length. It has been reported that eight weeks of EtOH plus corn oil feeding to the rats resulted in liver injury with hepatic macrophage activation, increased pro-inflammatory cytokine expression and neutrophil infiltration. These events were not observed in animals fed EtOH and dietary SF, specifically, cocoa butter (rich in long chain saturated fatty acids, palmitic acid (C16:0), and stearic acid (C18:0)) or MCTs (predominantly octanoic (C8:0), and decanoic (C10:0) acids) [7]. Further, endotoxemia was observed in response to EtOH plus corn oil, but not to either cocoa butter or MCTs. On the mechanistic level, MCTs prevented down regulation of intestinal TJ proteins, while cocoa butter normalized EtOH-increased hepatic endotoxin levels via up-regulation of an endotoxin detoxifying enzyme, argininosuccinate synthase 1 [7]. Another recent study has demonstrated that mice supplemented with saturated long chain fatty acids (LCFA, palmitic acid (C16:0, 25%), and stearic acid (C18:0, 85%)) developed less severe EtOH-associated liver disease, and these mice had reduced levels of hepatic injury, steatosis, and oxidative stress compared to mice fed EtOH and a diet rich in USF (oleic acid (C18:1, 27%), and linoleic acid (C18:2, 60%)) [8]. LCFA supplementation could stabilize the gut barrier, reduce EtOH-mediated intestinal inflammation, and reduce microbial translocation. The authors concluded that these are the factors contributing to the protective effects of LCFA against EtOH-induced liver injury.

## 3. Dietary PUFAs and ALD: The Bad, the Good, and the Controversy

**Figure 1:**
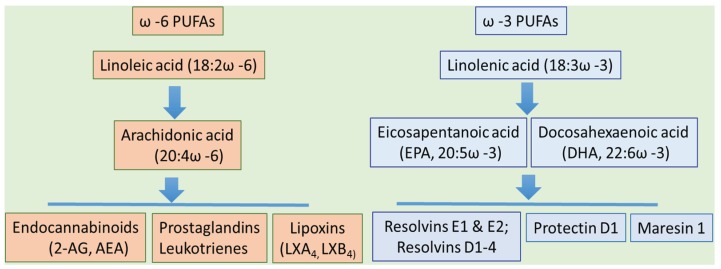
Omega ω-6 and ω-3 essential fatty acids and their metabolites. Abbreviations: AEA: *N*-arachidonoylethanolamine (anandamide); 2-AG: 2-arachidonoylglycerol; LXA_4_: lipoxin A_4_; LXB_4_: lipoxin B_4_; PUFA: polyunsaturated fatty acid.

It is important to note that not all dietary PUFAs play an equal role in ALD pathogenesis. There are two major families of dietary PUFAs, specifically omega-6 (ω-6) and omega-3 (ω-3) PUFAs, with numerous related metabolites (Figure 1). Although the large body of experimental evidence suggests that dietary LA, an omega-6 PUFA (18:2ω-6), exacerbates ALD [4,5,6,7,16], the role of omega-3 PUFAs, e.g., alpha linolenic acid (ALA, (18:3ω-3)), eicosapentaenoic acid (EPA, (20:5ω-3)) and docosahexaenoic acid (DHA, (22:6ω-3)) in ALD is not completely understood. In general, ω-3 PUFAs, specifically EPA and DHA, are known to have important biological effects on a range of cellular functions resulting in beneficial effects on health, including reduced risk of cardiovascular disease and inflammation [31,32,33,34,35]. It is increasingly recognized that the Western type diet is low in ω-3 PUFAs and rich in ω-6 PUFAs [36], which results in an increased ω-6/ω-3 PUFA intake ratio (Table 3). Dietary imbalance between ω-6 and ω-3 PUFAs leads to adverse health effects, including NAFLD [37,38]. There is evidence that ω-3 PUFA supplementation resulted in a reduced plasma ω-6/ω-3 ratio [39] and ameliorated hepatic steatosis in patients with NAFLD [40,41]. In rodents, ω-3 PUFA depletion promotes hepatic fat accumulation and insulin resistance [42], while ω-3 PUFA supplementation significantly decreased [43,44,45,46] or reversed high-fat diet induced liver steatosis [47]. Recent molecular and cellular advances provided new mechanistic insight into the role of specific PUFAs in liver pathology. For example, supplementation of highly purified EPA significantly ameliorated hepatic fat accumulation by inhibition of lipogenesis via suppressing SREBP-1 [45]. The combination of EPA and DHA either reduced or normalized to control values high fat diet-induced hepatic oxidative stress, steatosis (via PPARα up-regulation), and inflammation (via nuclear factor kappaB (NF-kappaB) DNA binding abrogation) [43,46].

The results regarding the role of dietary ω-3 PUFAs in ALD are somewhat inconsistent. It has been shown that a diet containing fish oil, specifically menhaden fish oil, promotes severe liver injury and inflammation in the EtOH feeding rat model [17,48]. These effects were attributed to markedly enhanced hepatic cytochrome P450 2E1 (CYP2E1) levels and lipid peroxidation. On the other hand, there are several studies demonstrating that fish oil or purified ω-3 PUFAs (e.g., EPA and DHA) are beneficial in ALD [49,50,51]. For example, prior ingestion of fish oil, specifically tuna fish oil (30% of total energy), reduced hepatic fat accumulation caused by a single dose of ethanol administration in mice, at least in part, through a marked reduction in hepatic stearoyl-CoA desaturase-1 (SCD-1) expression and SREBP-1 activity [49]. Another study reported that compared to the control (EtOH-alone) group, the mice supplemented with highly purified DHA had significantly decreased EtOH-induced serum alanine aminotransferase (ALT) activity, pro-inflammatory cytokine levels (interleukin 6 (IL-6) and tumor necrosis factor α (TNF-α)), and were protected against fat accumulation in the livers [51]. This study has also demonstrated that hepatic SCD-1 expression and activity were significantly reduced, whereas the expression of heme oxygenase-1 (HO-1), an enzyme that can improve cell survival in liver tissue, was markedly increased in DHA-supplemented mice compared to the control animals [51]. It has also been reported that rats fed a diet supplemented with physiologically relevant concentrations of ω-6 PUFAs, arachidonic acid (AA, (20:4ω-6)) and the ω-3 PUFA, DHA (AA:DHA = 1:1), were protected against EtOH-induced fatty liver and mitochondrial dysfunction, most likely through reducing oxidative/nitrosative stress [50]. The beneficial role of ω-3 PUFAs in ALD has been also supported by the observation that when rhesus monkeys were fed a diet that was generally nutritionally adequate (including the LA amount), but had a low ω-3 PUFA content (very low concentration of ALA) with a free access to an ethanol solution, they developed hepatic steatosis and fibrosis [52,53]. It could be speculated that the discordant results regarding the role of ω-3 PUFAs in experimental ALD may reflect the differences in the amounts used to supplement the experimental diets (e.g., large amount in the study demonstrating damaging effects [17] *vs.* small or physiologically relevant ω-3 PUFA quantities in the studies reporting protective effects [50]). Moreover, the fish oil source, with a variable content of EPA and DHA, may also play a role. In addition, the controversy might also be attributed to the nature of fish oil, which contains large amounts of highly unstable, easily oxidized *ex vivo* long-chain ω-3 PUFAs. It has been suggested that ingestion of lipid peroxidation products present in oxidized fish oil may cause negative health effects [54]; the oxidation status of the fish oil in the reviewed studies has not been considered. Lastly, the PUFA ω3/ω6 ratio in a diet needs to be taken into consideration as an important factor contributing to the beneficial or adverse health effects.

**Table 3:** Food sources and dietary intake of major ω-3 and ω-6 fatty acids *.

Fatty Acids	Food Sources	Dietary Intake
**ω3 PUFAs**		
α-Linolenic Acid, 18:3 ω-3	Vegetable oils (e.g., soybean and canola). Nuts, and seeds.	1.4 g/d
Eicosapentanoic acid, 20:5 ω-3	Fish (e.g., halibut, mackerel, herring, and salmon) and fish oils	0.1–0.2 g/d
Docosahexaenoic acid, 22:6 ω-3		
**ω6 PUFAs**		
Linoleic Acid, 18:2 ω-6	Vegetable oils (e.g., sunflower, safflower, soybean, corn, and canola), nuts, seeds meats, and eggs	12–17 g/d

* References: [55,56].

## 4. Oxidized Dietary Fat: Relevance to Liver Pathology

The Western diet contains large quantities of oxidized lipids, including oxidized fatty acids, oxidized cholesterol, cytotoxic aldehydes, and phospholipids that are formed during different cooking processes (e.g., using a variety of vegetable oils for deep frying, repeated heating) or long term storage [54,57,58,59]. Moreover, natural antioxidants (e.g., phenols, vitamin E, and other tocopherols) in the oils also deteriorate after heating [57,60]. There are numerous factors affecting the quality of oil during deep frying, including the type and quality of frying oil, replenishment of fresh oil, frying time and temperature, and food antioxidants [57]. It is not in the scope of this review to discuss the chemistry of lipid peroxidation, but rather to draw attention to the significance of dietary oxidized lipids in human health and disease [61,62], including their role in ALD pathogenesis. It should be mentioned that no dietary recommendations regarding the toxic or nontoxic amounts of oxidized lipids have been established.

Increasing evidence suggests that oxidized lipids from dietary heated oils contribute to numerous adverse health effects, such as postprandial oxidative stress and inflammatory response [60,63], and cardiovascular disease [62]. There are limited data regarding the effects of oxidized dietary lipids on the liver pathology. It has been reported that rats fed a diet rich in thermally-oxidized palm oil (150 °C, five rounds for 20 min each) developed more severe liver injury (assessed by significant increase in ALT levels) compared to animals fed a diet containing fresh palm oil [64]. Thermally-oxidized corn oil (180 °C for 18 h) also induced hepatic lipid peroxidation [65], and liver steatosis and injury in rats [66]. In regard to ALD, it has been shown that the rats, given EtOH and a diet containing thermally treated sunflower oil (180 °C, two rounds for 30 min) developed more severe liver injury compared to animals given either EtOH or thermally treated sunflower oil alone [67,68,69,70]. Liver injury in these animals was attenuated by *Phyllanthus niruri* (a potent natural antioxidant) leaf extract [71]. Conversely, another study demonstrated that feeding EtOH and a diet containing oxidized sunflower oil (60 °C for 25 d) compared to EtOH and a diet containing unheated oil resulted in markedly lower liver fat accumulation in rats via a mechanism involving an increase in the expression of PPARα target genes [72]. However, the effects of oxidized lipids on EtOH-mediated liver injury and inflammation in this animal model remain unclear as no data were provided. Future studies determining the role and underlying mechanisms of either harmful or potentially beneficial effects of oxidized dietary lipids on human health and disease, including liver pathology are warranted.

## 5. Oxidized Linoleic Acid Metabolites: Implication for ALD

**Figure 2:**
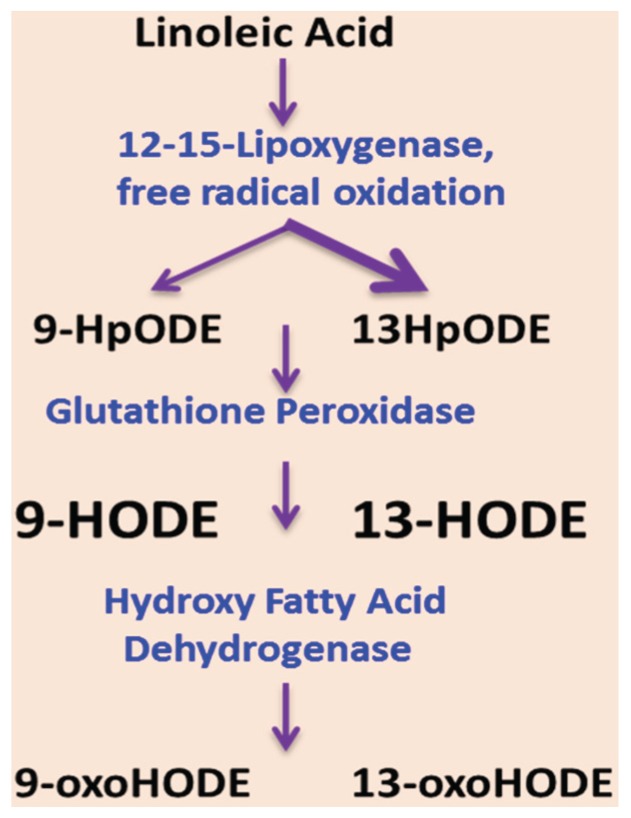
Oxidative metabolism of linoleic acid. LA can be enzymatically or non-enzymatically converted to 9- and 13-HpODE, with subsequent enzymatic conversion to hydroxy (9- and 13-HODE) and ketone (9- and 13-oxoODE) derivatives. Abbreviations: HpODE: hydroperoxy-octadecadienoic acid; HODE: hydroxy-octadecadienoic acid; oxoODE: oxo-octadecadienoic acid.

**Figure 3:**
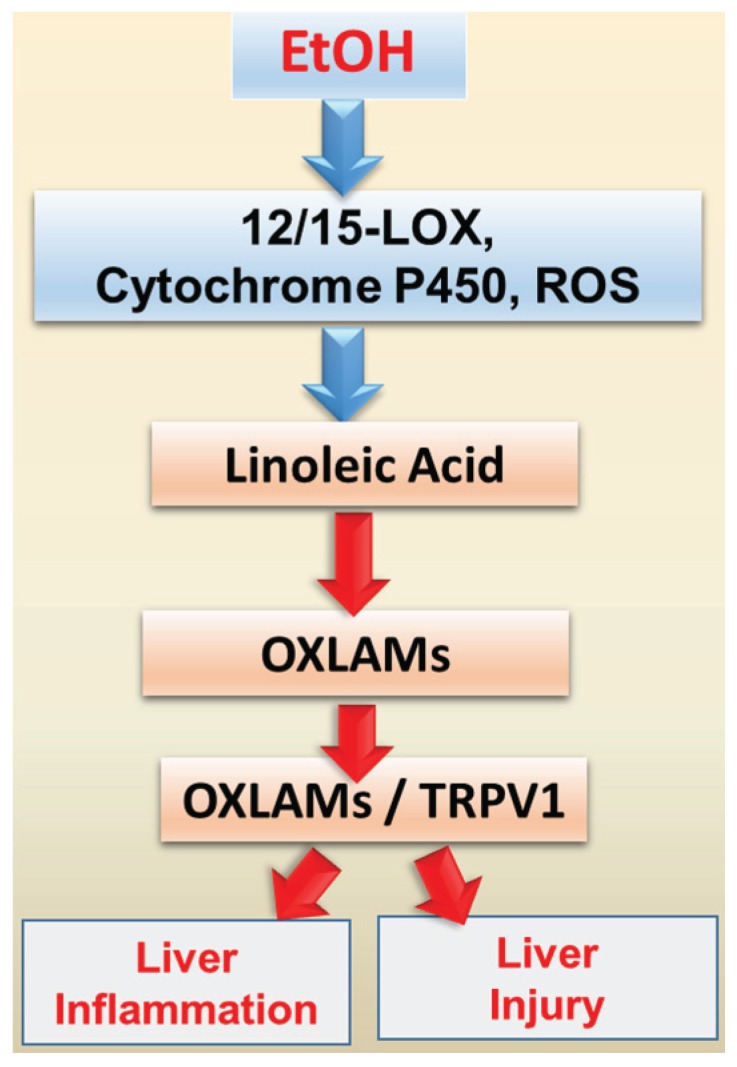
Proposed model of OXLAM/TRPV1-mediated mechanism of USF and EtOH-mediated liver injury and inflammation. Abbreviations: EtOH: ethanol; LOX: lypoxygenase; ROS: reactive oxygen species; OXLAMs: oxidized linoleic metabolites; TRPV1: Transient Receptor Potential Vanilloid 1.

Given that: (i) dietary LA is required for the development of experimental ALD [16]; (ii) LA is a major unsaturated fatty acid in the Western diet [55]; and (iii) LA consumption has dramatically increased during the 20th century with the soybean oil, poultry, and shortening as the primary dietary LA sources [36], it is important to determine the mechanisms underlying the deleterious effects of dietary LA on EtOH-mediated liver injury. Based on several observations made in NAFLD and ALD, in both clinical and pre-clinical studies, a new concept has recently emerged that bioactive oxidized linoleic metabolites (OXLAMs), which are formed enzymatically from LA primarily via the actions of 12/15-lipoxygenase (12/15-LOX), or non-enzymatically via free radical-mediated oxidation in response to oxidative stress (Figure 2), might contribute to ALD pathogenesis. It has been demonstrated that OXLAMs, specifically 9- and 13-hydroxy-octadecadienoic acids (9- and 13-HODEs), were selectively elevated without corresponding increases in oxidation products of other fatty acids in patients with nonalcoholic steatohepatitis [73], and decreases in plasma OXLAM levels correlated with hepatic histological improvement [74]. Elevated plasma 9- and 13-HODEs levels in patients with alcoholic cirrhosis were observed in parallel with the increase in lipoxygenases (15-LOX-1 and 15-LOX-2 mRNA) in the liver samples [75]. Notably, the plasma content of HODEs in patients with ALD was more than 46 times higher than in healthy subjects and more than four times higher than in NAFLD patients [75]. Further, increased levels of 9- and 13-HODEs were observed in experimental animal models of ALD [76,77] in parallel with the hepatic steatosis, oxidative stress and hepatocyte damage. It has been reported that 9- and 13-HODEs are natural endogenous ligands for the Transient Receptor Potential Vanilloid 1 (TRPV1) [78,79]. Our recent study demonstrated that chronic-binge EtOH-mediated increases in circulating OXLAMs and TRPV1 levels in mice were associated with hepatic steatosis, inflammation, and injury [77]. Importantly, we found that TRPV1 deficiency protected against chronic-binge alcohol induced hepatic inflammation and injury with no effects on hepatic steatosis suggesting that OXLAM/TRPV1 interactions may contribute to the progression from the simple steatosis to steatohepatis (Figure 3). The detailed mechanism(s) remain to be determined. Taken together, OXLAMs might be the metabolites underlying pathogenic effects of dietary USF on EtOH-mediated liver injury via OXLAM/TRPV1-mediated or other mechanism(s); this hypothesis needs to be investigated further.

## 6. Dietary Fat and EtOH-mediated Changes in the Gut Microbiota

Over the past decade, the intestinal microbiota have been increasingly recognized as a critical factor in the pathogenesis of ALD. The gastrointestinal microbiome is composed of trillions of organisms [80], which perform a diverse range of metabolic functions [81], including production of numerous metabolites that serve as the nutritional source for microbes as well as important messengers between the microbiota and the host [82]. Several recent studies have shown that alcohol consumption is associated with alterations in the gut microbiota community, changes in microbial metagenome (collective genome of the bacterial community) and the metabolome (small molecule metabolites produced by microbiota), which may contribute to the dysregulated gut-liver axis, thereby exacerbating alcohol-induced liver inflammation and injury [8,83,84,85,86,87,88,89]. Both alcohol and diet play pivotal roles in ALD pathogenesis, therefore it is important to consider that the dietary patterns and dietary factors *per se* play a critical role in shaping the gut microbiota [90,91,92,93,94,95,96]. Diet can have a significant impact on the gut environment (e.g., gut transit time and pH), and intakes of major macronutrients (carbohydrates, proteins and fats) can significantly affect the microbiota composition. For example, a recent study reported that the Western diet, which is a high saturated fat/high-sugar diet, substantially affects the human microbiome composition and metabolic function [96]. The effects of specific dietary fat on the gut microbiome have been demonstrated in animal models; for example, a high fat saturated diet containing palm oil (palmitic and oleic acids are the major components) reduced microbial diversity and increased the *Firmicutes/Bacteroidetes* ratio [93]. Another study reported a decrease in *Bacteroidetes* and an increase in both *Firmicutes* and *Proteobacteria* associated with switching to the high saturated fat diet containing lard (pork fat; ≈40% saturated fatty acids, 45% monounsaturated fatty acids) [90]. There are few studies investigating the effects of different types of dietary fat on the alcohol-mediated changes in the gut microbiota. A recent study from our group demonstrated that chronic EtOH and USF (corn oil/LA rich) feeding in mice caused a decline in the abundance of both *Bacteriodetes* and *Firmicutes* phyla, with a proportional increase in the gram-negative *Proteobacteria* and gram-positive *Actinobacteria* phyla; these events were associated with disruption of the intestinal barrier, endotoxemia, liver steatosis, inflammation, and injury [86]. These microbiota alterations, and pathological changes in the intestine and the liver were prevented in EtOH and SF (MCT rich diet) fed mice (Kirpich I. *et al.*, [97]). It has also been reported that EtOH and a diet supplemented in saturated LCFA (rich in palmitic and stearic acids) maintained intestinal eubiosis compared to mice fed EtOH and USF diet (rich in oleic acid and LA), in which the microbial dysbiosis characterized by reduced proportion of the *Firmicutes*, increased numbers of *Bacteriodetes*, and reduced proportion of *Lactobacillus* species [8]. Notably, the USF + EtOH induced microbial dysbiosis was associated with intestinal barrier dysfunction and liver injury, which were prevented by LCFA supplementation. The authors further determined that EtOH-mediated intestinal dysbiosis resulted in reduced capacity of the microbiome to synthesize saturated LCSFs that are essential for growth of *Lactobacillus*, which appears to produce factors that promote proper intestinal barrier function [8]. To summarize, chronic alcohol consumption is accompanied by alterations of gut microbiota composition and metabolic functionality, and dietary factors likely play a critical role in shaping of these changes. Further research is required to define the interactions between the specific types of dietary lipids, the gut microbiota, and ALD development and progression.

## 7. Therapeutic Implications of Dietary Lipids in ALD

If validated in human studies, the cumulative experimental dietary data have important implications for ALD including prevention and treatment. Dietary factors such as specific unsaturated fats may help explain why only some people who drink heavily develop progressive ALD. Dietary restriction of potentially “harmful” lipids could help prevent ALD in those who drink heavily (although this is probably unrealistic). For patients with ALD who are prescribed or take nutritional supplements, the optimal lipid composition may include MCTs (indeed, they are already used in many enteral products). The American Society of Parenteral and Enteral Nutrition identified MCT oil as a potentially beneficial additive to the lipid emulsions for the parenteral nutrition [98]. “Nutritional” drugs such as tributyrin (prodrug of butyrate, a short chain fatty acid) may be beneficial in ALD. Indeed, a pre-clinical experimental study has recently demonstrated that tributyrin prevented short-term EtOH-induced increases in ALT and hepatic pro-inflammatory cytokine and chemokine expression, and protected mice from acute ethanol-induced gut injury [99]. Thus, there are multiple “lipid interventions” that may prove beneficial in ALD.

## 8. Conclusions

**Figure 4:**
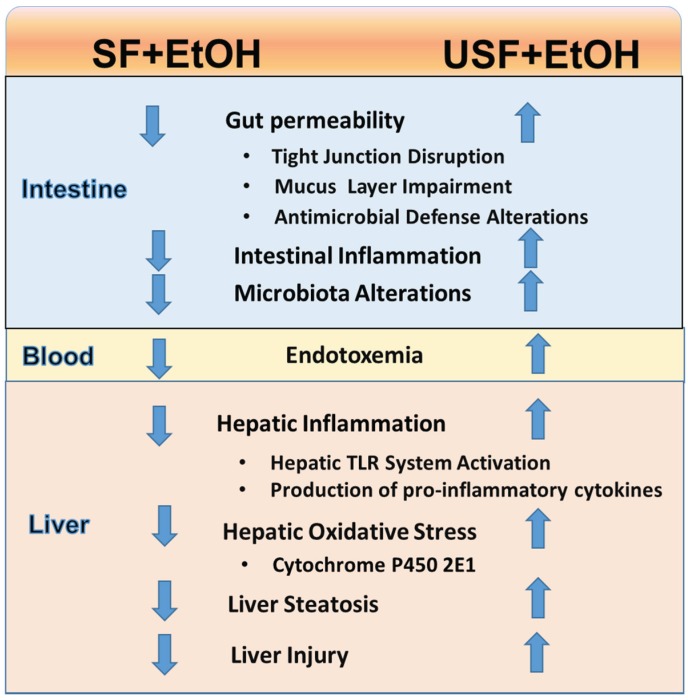
Differential effects of dietary saturated and unsaturated fat on EtOH-induced intestinal and liver alterations.

In conclusion, there is convincing experimental evidence demonstrating the differential effects of different types of dietary lipids in the pathogenesis of ALD. While the protective effects of dietary SF and deleterious effects of dietary USF (primarily rich in LA, an ω-6 PUFA) on ethanol-induced intestinal and liver injury have been well documented in animal models (Figure 4), the effects of dietary ω-3 PUFAs as well as the significance of the dietary PUFA ω-3/ω-6 ratio in ALD development and progression are not completely understood. The role and the significance of oxidized lipids, both dietary and *in vivo*-produced, as well as possible mechanisms underlying their beneficial or deleterious effects in liver pathology remain to be determined. Different types of dietary fat may differentially modulate the gut-liver axis, including EtOH-induced changes in the gut microbiota. This important aspect needs to be considered when evaluating the effects of EtOH consumption on the gut microbiota composition and function. Given that there is no FDA-approved therapy for any stage of ALD, dietary fat may play an important role in the management of ALD.

## Abbreviations

AA	arachidonic acid
AEA	*N*-arachidonoylethanolamine (anandamide)
2-AG	2-arachidonoylglycerol
ALA	alpha linolenic acid
ALD	alcoholic liver disease
ALT	alanine aminotransferase
AMPK	AMP-activated protein kinase
CYP2E1	cytochrome P450 2E1
DHA	docosahexaenoic acid
HNF4α	hepatocyte nuclear factor-4α
HO-1	heme oxygenase-1
HpODE	hydroperoxy-octadecadienoic acid
9- and 13-HODEs	9- and 13-hydroxy-octadecadienoic acids
EPA	eicosapentaenoic acid
EtOH	ethanol
IL-6	interleukin 6
LA	linoleic acid
LPS	lipopolysaccharide
12/15-LOX	12/15-lipoxygenase
LCFA	long chain fatty acids
LXA_4_	lipoxin A_4_
LXB_4_	lipoxin B_4_
MCT	medium chain triglyceride
NAFLD	nonalcoholic fatty liver disease
NF-kappaB	nuclear factor kappaB
OXLAMs	oxidized linoleic acid metabolites
oxoODE	oxo-octadecadienoic acid
PPARα	peroxisome proliferator-activated receptor alpha
PGC-1α	peroxisome proliferator-activated receptor gamma, coactivator 1 alpha
PUFA	polyunsaturated fatty acid
ROS	reactive oxygen species
SF	saturated fat
SCD-1	stearoyl-CoA desaturase-1
SIRT1	Sirtuin
SREBP-1	sterol regulatory element binding protein-1c
TJs	tight junctions
TG	triacylglycerol
TLR	Toll-like receptors
TRPV1	Transient Receptor Potential Vanilloid 1
TNF-α	tumor necrosis factor α
USF	unsaturated fat
